# Multi-omic network signatures of disease

**DOI:** 10.3389/fgene.2013.00309

**Published:** 2014-01-07

**Authors:** David L. Gibbs, Lisa Gralinski, Ralph S. Baric, Shannon K. McWeeney

**Affiliations:** ^1^McWeeney Lab, Division of Bioinformatics and Computational Biology, Oregon Health & Science UniversityPortland, OR, USA; ^2^Baric Lab, Department of Microbiology and Immunology, University of North Carolina at Chapel HillChapel Hill, NC, USA; ^3^McWeeney Lab, OHSU Knight Cancer Institute, Oregon Health & Science UniversityPortland, OR, USA

**Keywords:** omics, networks, data integration, proteomics, transcriptomics, virology, biomarkers, SARS

## Abstract

To better understand dynamic disease processes, integrated multi-omic methods are needed, yet comparing different types of omic data remains difficult. Integrative solutions benefit experimenters by eliminating potential biases that come with single omic analysis. We have developed the methods needed to explore whether a relationship exists between co-expression network models built from transcriptomic and proteomic data types, and whether this relationship can be used to improve the disease signature discovery process. A naïve, correlation based method is utilized for comparison. Using publicly available infectious disease time series data, we analyzed the related co-expression structure of the transcriptome and proteome in response to SARS-CoV infection in mice. Transcript and peptide expression data was filtered using quality scores and subset by taking the intersection on mapped Entrez IDs. Using this data set, independent co-expression networks were built. The networks were integrated by constructing a bipartite module graph based on module member overlap, module summary correlation, and correlation to phenotypes of interest. Compared to the module level results, the naïve approach is hindered by a lack of correlation across data types, less significant enrichment results, and little functional overlap across data types. Our module graph approach avoids these problems, resulting in an integrated omic signature of disease progression, which allows prioritization across data types for down-stream experiment planning. Integrated modules exhibited related functional enrichments and could suggest novel interactions in response to infection. These disease and platform-independent methods can be used to realize the full potential of multi-omic network signatures. The data (experiment SM001) are publically available through the NIAID Systems Virology (https://www.systemsvirology.org) and PNNL (http://omics.pnl.gov) web portals. Phenotype data is found in the supplementary information. The ProCoNA package is available as part of Bioconductor 2.13.

## Introduction

Statistical and computational methods are used in systems biology to infer underlying networks associated with disease (Aderem et al., 2011). Networks can be used for deriving predictive signatures of disease progression or severity, as well as helping to elucidate the underlying mechanisms (Zak and Aderem, 2009). A primary objective in systems biology is to understand the structure and connection between the diverse biological elements composing the living system, and how they dynamically change and interact in response to biologically important events, such as the host response to infection (Forst, 2006).

Single data-type signatures and biomarkers have found mixed success where many potentially useful biomarkers have not been validated (Ntzani and Ioannidis, 2003; Feng et al., 2004; Brenner and Normolle, 2007; Hughes, 2009; Bhavsar et al., 2010; Kint et al., 2010; Sturdevant et al., 2010). In virology, biomarkers could be used to predict the host response, allowing for earlier care, before the onset of extreme and damaging cytokine responses (Davey et al., 2013). The biomarker discovery process can utilize a range of different data types including genomic (DNA sequence data), transcriptomic (gene expression), proteomic (protein levels), metabolomics (metabolite levels), and prior biological knowledge such as that found in interactomics (encompassing protein-protein interactions databases).

It is thought that predictors or biomarkers utilizing multiple data types and/or exploiting the underlying network structure will prove more robust, as these more reflect the complex biology involved (Sung et al., 2012). A range of integration techniques have been suggested including machine learning methods (Lanckriet et al., 2004; Zhang et al., 2006; Daemen et al., 2008), probabilistic networks (Hartemink et al., 2002; Troyanskaya et al., 2003; Gat-Viks et al., 2006; Vaske et al., 2009), correlation networks (Adourian et al., 2008), statistical models (Nie et al., 2006; Fagan et al., 2007; Lê Cao et al., 2008; Torres-García et al., 2009), clustering techniques (Cancer Genome Atlas Network, 2012; Waters et al., 2012) and applications of spectral theory (Berger et al., 2006; Tan et al., 2009; Kim et al., 2012). To produce integrated network signatures, however, methods must be applied across extremely heterogeneous sources, which has proven difficult because of the extreme differences between data types. In particular, the integration of the transcriptome and proteome is a current challenge in omics research due to differences in dynamic range of measurements, incomplete annotation, isoform differences, and temporal effects, as several examples (Cox et al., 2005; Waters et al., 2006a,b; Cancer Genome Atlas Network, 2012).

**Figure 1:**
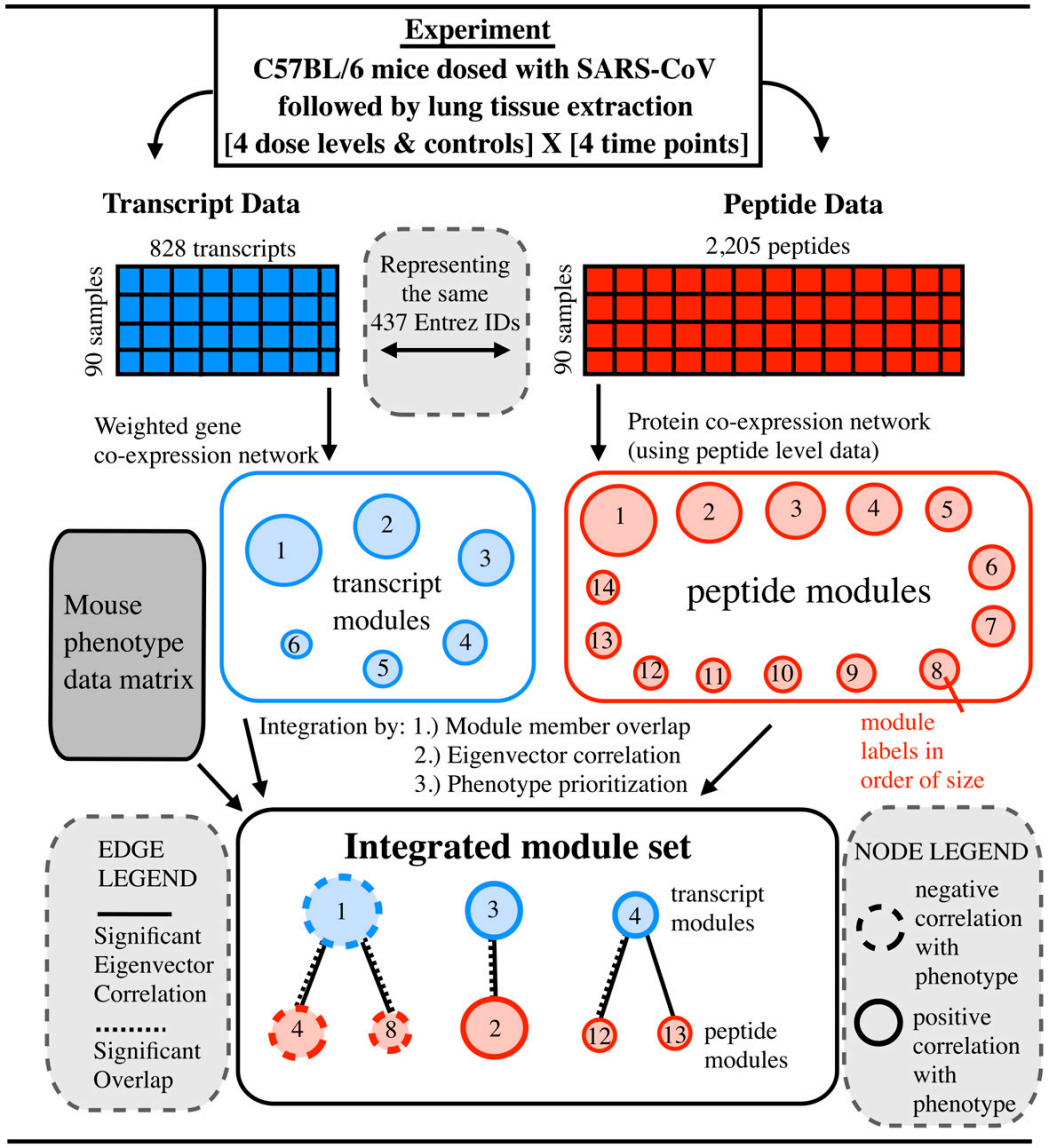
**Prioritization of integrated data leading to a multi-omic integrated co-expression signature for SARS-CoV infection**. Given mice infected with a mouse adapted SARS-CoV virus, transcript (gene microarray) and peptide (LC-MS) data are collected from lung tissue. The data is used to construct independent co-expression networks for each data type. Using three metrics, transcript and peptide modules are compared and joined, producing a bipartite module graph. In the module graph, two kinds of edges are shown. Solid edges indicate a significant correlation between module eigenvectors. Dashed edges indicate significant overlap in terms of module membership. Node sizes correspond to the size of the module and node outlines show the direction of correlation compared to the degree of lung pathology (the overall-pathology-score).

We have developed the methods needed to explore whether a relationship exists between co-expression network models built from transcriptomic and proteomic data types, and whether this relationship can be used to improve the disease signature discovery process. This work uses publically available data from an NIAID systems biology consortium study involving infection of SARS-CoV in mice. We have developed an approach to produce integrated network signatures of disease by leveraging earlier work on co-expression transcriptome networks (Zhang and Horvath, 2005; Yip and Horvath, 2007; Mason et al., 2009; Langfelder et al., 2008, 2011; Langfelder and Horvath, 2012) and our own work in proteomic co-expression networks (Gibbs et al., 2013). The signature consisted of a bipartite module graph, connecting co-expression modules obtained from transcriptomic and proteomic data, that is constructed using significant module member overlap, correlation of eigenvector summaries, and common phenotypic associations with outcomes of interest. The functional enrichment of module sub-graphs was overlapping across data types, further offering evidence of the underlying biological network structure. This work provides a framework for multi-omic prioritization of module members for biomarker studies as well as perturbation and validation experiments (see Figure 1).

## Methods

### Experimental data

This publically available data (https://www.systemsvirology.org, experiment SM001) was generated from 20-week-old C57/B6 mice infected with the MA15 mouse adapted SARS-CoV virus (Roberts et al., 2007). In total, transcript and proteomic expression profiles were collected for 92 mice representing four dosage levels (10^2^, 10^3^, 10^4^, 10^5^ PFU) over four time points (1, 2, 4, 7 days), including 3 mock samples per day (5 mice * 4 time points * 4 dose levels + 3 mocks * 4 time points).

The control mouse at Day 7, replicate 2, and infected mouse (PFU 10^2^), Day 4, replicate 3, were removed from the study since in the transcript data, the mice clustered with the incorrect infection label.

Phenotype data quantified the pathological severity resulting from infection (see Gibbs et al., 2013 for more on the phenotype data. The phenotype data can be found in Supplementary Table 3). While the difference in pathology level among the viral dosages is small, there was some observed difference in the kinetics of infection. Higher doses prompted more immediate responses. The difference in pathology among the viral dosages is relatively small. In total, 15 phenotype variables were recorded including an aggregate measure called the “overall pathology score.” Many of the phenotype variables are highly correlated, such as inflammation, airspace inflammation, and interstitial inflammation. Other phenotype variables include physical characteristics such as diffuse alveolar damage (DAD), debris, edema, and hyaline membranes. Day and dose are also included in the analysis.

### Taking the peptide-transcript intersection

Using the VIPER software (v3.48) (Monroe et al., 2007) peptides are matched to an Accurate Mass and Time (AMT) tag database (Zimmer et al., 2006). Details are given on the systemsvirology.org site and in Gibbs et al. (2013). Abundance measurements for 16,890 peptides mapping to 3277 proteins were recorded for 184 LC-MS runs. Taking all observed peptides, protein inference was performed using the Fido protein inference model (Serang et al., 2010; Serang and Noble, 2012). Inferred Proteins were accepted with scores above 0.95. From the total set of proteins, 691 proteins had this score or better.

Peptide data was filtered by peptide matching quality scores (given by the VIPER software) STAC (>0.6), UP (>0.5), and Peptide Prophet tag score (>0.9), which resulted in a matrix of 184 sample rows by 9326 peptides. Sample replicates were combined by taking the mean over replicates.

A second round of filtering was performed by assessing the quantity of missing data. Missing data are encountered when peptides are identified in a subset of samples. “Missingness filtration” involves removing any peptide with greater than X% missing data across samples. Peptides were filtered by missingness, taking peptides with not more than 20% missing data, resulting in a matrix of 90 samples by 2273 peptides. This process eliminates a large proportion of the measured peptides (86.5%), however, for statistical analysis that depends on nearly complete matrices, many peptides are unusable since they are identified in a very small number of samples.

The matched microarray data were processed using the Agilent Preprocess Bioconductor package (Lopez-romero, 2010). After considering quality measures, 31,416 probes passed probe QC flags for all replicates of at least one infected time point.

In order to focus on the dynamic relationship between the transcriptome and proteome, the two data types were subset using the intersection based upon Entrez gene IDs. Transcript probes on the gene microarray were mapped to Entrez gene IDs using annotation databases (mgug4122a.db) found in Bioconductor (2.11). Proteins in the AMT tag database were mapped to Entrez IDs and protein families using the Uniprot web service (Apweiler et al., 2004; Wu et al., 2006; Magrane and Consortium, 2011).

Networks were constructed using intersection between quality filtered peptide and transcript data. This corresponded to 90 total samples with 2205 peptides mapping to 445 Uniprot IDs (in the mass tag database) and 490 Entrez gene IDs. These gene IDs were used to select transcript probes, resulting in 814 probes that corresponded to 439 Uniprot IDs and 447 Entrez gene IDs. Between the two data sets, 437 Entrez IDs are shared. Some discrepancy was observed, since peptides are often degenerate, mapping to multiple proteins, creating a scenario in which an entry maps from a given Uniprot ID to multiple Entrez IDs. To address mapping discrepancies, after mapping to Entrez IDs, only IDs contained within the intersection were considered.

### Constructing the integrated bipartite module graphs

Network based integration of transcript and peptide data was performed by constructing independent co-expression networks for each data type using methods derived from WGCNA. (Langfelder and Horvath, 2008; Mason et al., 2009; Iancu et al., 2012a). Co-expression networks are composed of nodes connected by weighted edges. Edge weights were computed using signed, robust correlations producing a similarity matrix (Langfelder et al., 2012). The similarity matrix raised to a power, beta, produces the adjacency matrix. Beta was selected according to scale-free model criterion (R^2^ describes the model fit), peptide networks had an R^2^ greater than 0.8 and transcript networks greater than 0.9. The adjacency matrix is used to compute topological overlap between nodes, weighting the network edges. Topological overlap is defined as TOM_ij_ = (l_ij_ + a_ij_) / [min(k_i_, k_j_) + 1 − a_ij_] where l_ij_ is the dot product on row i and column j in adjacency matrix [a] and k_i_ (the connectivity) is the summation of row i in adjacency matrix [a].

Groups of nodes were partitioned into subnetworks, or modules, containing (in this case) either transcripts or peptides. Modules were composed of strongly connected (high edge weights) nodes. The dynamic hybrid treecut method was used to derive subnetworks or modules (Langfelder et al., 2008), using default settings.

Modules were summarized by taking the first right-singular vector produced by singular value decomposition on expression data represented by nodes contained in the module. The module eigenvectors (MEs or module summaries) allowed us to associate modules to biological phenotypes using correlation. Modules were labeled numerically according to decreasing size, module 1 being the largest.

Although the hybrid treecut algorithm generally works well, there is no guarantee for a module's average connectivity to be greater than what is expected by random. Permutation testing was used to assess module significance by comparing the mean topological overlap of nodes within a module to the mean topological overlap of a randomly sampled set of nodes equal in size to the module being tested. This is equivalent to permuting the module labels on nodes. Ten thousand permutations were performed.

Finally, to build the bipartite module graph, the first step involved measuring the member overlap between all pairs of modules (peptide-transcript). To test the significance of overlaps, random modules were constructed, keeping the module sizes fixed (equivalent in size to our derived modules) and varying the contents. Ten thousand permutations were performed. The count of permuted overlaps larger than the observed was used as an empirical *p*-value. Significant overlaps were used as edges between modules if FDR adjusted *p*-values were less than 0.1. This relaxed threshold was picked to increase sensitivity.

The edges of the bipartite graph are filtered by correlation between eigenvector summaries. The connection between module eigenvectors was measured using the Pearson correlation. Using Bonferroni multiple testing correction, *p*-values less than 0.0006 [0.05/(14 peptide modules * 6 transcript modules)] were accepted. If deemed appropriate, edges could be retained without significant overlap, as long as the annotation reflects that. The third step involved filtering edges by comparing the joined modules association to a phenotype. Edges were kept if the eigenvector-phenotype association was in the same direction and adjusted *p*-values were less than 0.05.

The combination of these three measures—member overlap, eigenvector correlation, and similar phenotype associations—constructed an integrated bipartite module graph that describes an integrated signature.

Functional enrichment via Gene Ontology terms was performed using the GOstats package (Ashburner et al., 2000; Falcon and Gentleman, 2007). The universe consisted of the 5521 Entrez IDs found in mass tag database, the limiting factor on peptide identifications. The conditional method was used which minimizes the correlation between GO terms. *P*-values were adjusted using the Benjamini and Yekutieli method (Benjamini and Yekutieli, 2001).

### Naïve method based on correlation for comparison to the module level analysis

A Pearson correlation based naïve approach was designed for comparison to the module level analysis. The approach involved computing correlations—independently for each peptide and transcript—on the overall pathology score phenotype. This produced two ranked lists for peptides and transcripts, each with both positive and negative correlations. The naïve top ranked entities were compared to the rankings within modules.

For comparison to the enrichment results, members of the ranked lists were selected from the most negative and most positive correlations separately, with size equal to the mean module sizes for the peptide and transcript networks (151 peptides and 130 transcripts). These selections were used for gene ontology enrichment using the same method as described in Constructing the Integrated Bipartite Module Graphs.

## Results

### Characterization of the co-expression networks obtained from single omic data types

The transcript network consisted of 6 modules containing between 42 and 357 transcripts. The peptide network consisted of 14 modules containing between 70 and 316 peptides. The modules were labeled numerically in order of decreasing size. Each co-expression network independently showed significant Pearson correlations between the module eigenvectors and phenotype data (See Supplementary Figures 1 and 2). In both networks, the greatest positive correlation was found with day of infection (transcript module 3, *r* = 0.84, peptide module 10, *r* = 0.70). When inflammation related variables were considered (inflammation, airspace inflammation and interstitial septum inflammation) transcript module 3 and peptide module 2 showed the strongest correlations (inflammation, transcript module 3, *R* = 0.6, peptide module 2, *R* = 0.6). These modules also showed the strongest correlation with the overall pathology of the mice. Conversely, transcript module 1 and peptide module 4 showed the same pattern of associations with phenotype as transcript module 3 and peptide module 2, but with negative correlations. Members of these modules had abundance profiles that decreased over time. In contrast to the previous two patterns of association, transcript module 4 and peptide module 12 were more associated with the administered viral dosage instead of day of infection. The modules showed strong associations with denudation, debris, and airway pathology whereas the previous two module sets did not.

### Initial construction of the bipartite module graph by overlap of module members

Significant module member overlaps were observed between the two co-expression networks. Overlaps were represented as a count of similar Entrez gene IDs (after mapping). Using permutation testing we ascertained whether the size of the overlap was larger that what is expected by chance. Permutation test significance was defined as FDR adjusted *p*-values less than 0.1. By that definition, ten out of 84 possible significant module overlaps were observed, forming three distinct sub-graphs, initializing the bipartite module graph. Overlaps were quantified by taking |Intersection(A,B)|/ min[size(A), size(B)]. Transcript module 1 overlapped with peptide modules 1, 4, and 8 (overlap amounts of 0.33, 0.39, and 0.42 with FDRs 0.0, 0.08, and 0.016 respectively). Transcript module 2 overlapped with peptide modules 2 and 3 (overlap amounts of 0.38 and 0.35 with FDRs 0.016 and 0.041 respectively). Transcript module 3 overlapped with peptide modules 2, 3, 5, and 10 (overlap amounts of 0.37, 0.38, 0.31, and 0.26 with FDRs of 0.068, 0.016, 0.087, and 0.016 respectively), and lastly, transcript module 4 overlapped with peptide module 12 (overlap amount 0.11, FDR 0.080). These overlaps formed the initial edges of the bipartite module graph.

### Module eigenvector correlation confirms and adds edges to the bipartite graph

From the 10 edges in the overlap graph, eight showed significant eigenvector correlation after Bonferroni multiple testing correction (*p*-values < 0.0006; See Supplementary Figure 3). Summarizing the results: the Pearson correlations between transcript module 1 and peptide modules 4 and 8 were 0.523 and 0.434 respectfully (*p*-values 1.16e-07 and 1.908e-05). The correlation between transcript module 4 and peptide modules 12 and 13 was 0.696 and 0.683 (*p*-values 1.159e-13 and 2.554e-14). The correlation between eigenvectors of transcript module 3 and peptide modules 2 and 10 was 0.755 and 0.801 (*p*-value 2.2e-16 for both). A particularly interesting case is seen with transcript module 4 and peptide module 13, where module overlap is not observed, but a strong connection between module eigenvectors is present. This encouraged us to include an additional edge between these modules. These cases are potentially very interesting connections, where connected modules are driven by unmatched hubs in the network, which might imply previously unknown interactions.

### Additional edge confirmation with shared phenotype associations

The bipartite module graph can be further modified by comparing independent module associations to a phenotype of interest. Strong correlation between eigenvectors typically brings a shared correlation to sample phenotypes due to the similar vector structures. However, correlation is not transitive, which explains why this should be accounted for, because if two module eigenvectors correlate, it does not mean the two modules both correlate to a given phenotype.

Considering only the overall pathology score phenotype, transcript module 3 and peptide module 2 remained connected. Transcript module 4 and peptide modules 12 and 13 also demonstrated strong shared phenotype associations. Transcript module 1 and peptide modules 4 and 8 shared negative correlations with the overall pathology. These associations reinforced the bipartite graph structure. Edges that did not reflect this similarity in phenotype associations were removed.

**Figure 2:**
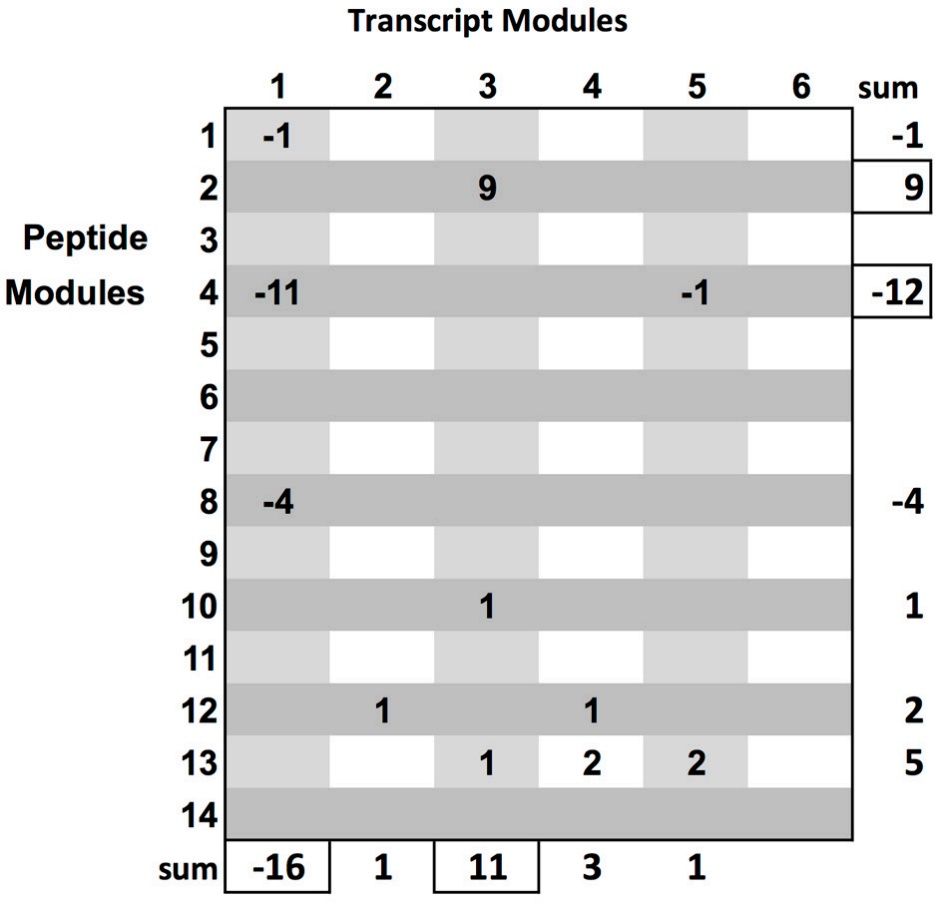
**Module graph prioritization by examination of the relationship of module-pairs to all phenotypic variables**. Clear patterns show transcript module 1 and peptide module 4 with the bulk of maximum negative correlations and transcript module 3 and peptide module 2 with the bulk of maximum positive correlations with the phenotype. This matrix clearly provides ranking on sub-graphs.

The rich set of phenotypes was used to prioritize the bipartite sub-graphs. We briefly describe an algorithm to prioritize module sub-graphs, given a set of phenotypes: initialize an n by m matrix where n is the number of transcript modules and m is the number of peptide modules. For each phenotype, the maximum and minimum (i.e., negative correlation) correlating modules from each data type are found. For each pair of modules in the matrix, and for each phenotype, if a pair of modules is maximum, +1 is added to the matrix element corresponding to this pair, and if the module pair has the minimum correlation, a −1 is added to the matrix position corresponding to the pair (see Figure 2 and Supplementary_Network_Results). In some cases, a module could arrive at a final summed score of 0 by alternatively winning both negative and positive correlations. Therefore, it might be necessary to keep scores strictly positive. This would return the maximum score by magnitude, without regard to direction, as an alternative prioritization procedure. After the prioritization routine, the set of biologically relevant multi-omic modules is ranked, providing a clear path toward targeted, downstream, analysis.

### Module sub-graphs show temporal trends

**Figure 3:**
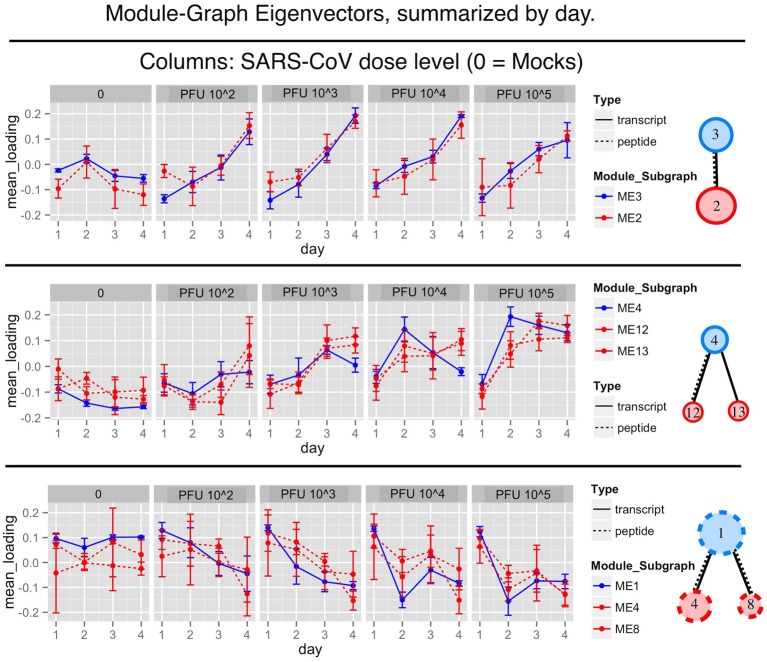
**Overlapping eigenvector summaries show similar response patterns observed across transcript and peptide modules**. The blue lines show collapsed transcript module eigenvectors plotted over days post infection. Red dotted lines show the collapsed peptide module eigenvectors. The top row shows the module sub-network of transcript module 3 and peptide module 2. The middle row shows transcript module 4 and peptide modules 12 and 13. The bottom row shows transcript module 1 with peptide modules 4 and 8. There is clear evidence of a shared response between transcript and peptide modules, demonstrating a multi-omic signature. See the supplementary tables for lists of the drivers and enriched functional categories.

The peptides and transcript expression profiles, within a module sub-graph, showed two types of temporal patterns. The expression response either varied with time or with viral dosage. The patterns are made clear after collapsing the eigenvector summaries by day (see Figure 3).

The first row of Figure 3 shows the abundance peaks of transcript module 3 and peptide module 2. Baseline abundance is observed on day 1 followed by abundance increases over the course of infection.

The second type of trend is exemplified by transcript module 1 with peptide modules 4 and 8, as well as transcript module 4 with peptide modules 12 and 13. This trend showed a response pattern associated with viral dosage rather than time. This is clearly seen in the high dose column. In summary, we have found two different patterns of module response, one by time (increasing over time) and the other by dosage.

### Sub-graphs share functional annotations

After sub-graph prioritization, we were interested in what biological entities were most central (i.e., correlated with the module eigenvector) in the network modules. By filtering module members by centrality, we have a metric by which to rank and prioritize the module members. Taking the ten most central module elements and using Uniprot web services, we examined the associated protein families. For transcript module 1 and peptide module 4, shared families included the Caveolin family, GST superfamily, Mu family, Cu-Zn superoxide dismutase family and transcript module 4 contained members of the aldehyde dehydrogenase family. These protein families are associated with metal binding proteins, interactions with DNA and engaging in changes to acetylation patterns. Putative caveolin-binding sites have been observed in SARS-CoV encoded proteins, and aldehyde dehydrogenases have been found to have a role in infection (Cai et al., 2003; Cinatl et al., 2004).

In transcript module 3, central members included proteins from the CAP family, Histone H1/H5 family, Histone H2A family, and the intermediate filament family, while for peptide module 2 protein families included the GTP-binding elongation factor family, the EF-Tu/EF-1A subfamily, the heat shock protein 90 family, the Histone H1/H5 family, and the DEAD box helicase family (eIF4A subfamily). Both histones and elongation factors have previously been associated with SARS-CoV infection (Reghunathan et al., 2005).

Transcript module 4 and peptide modules 12 and 13 were enzyme-driven and were associated with Serpin family members, which are protease inhibitors. This is potentially important since it was recently reported that—along with Serpin1—the coagulation and urokinase pathways are activated during infection. (Gralinski et al., 2013). This is closely related to lung pathology involving disseminated small vessel thromboses in the lungs (Ng et al., 2004a,b; de Lang et al., 2007). This module sub-graph could be used for further examining the systems level connection between omics and pathology.

Examination of significant GO terms shows largely similar trends compared to protein family annotations (all adjusted *p*-values are Bonferroni adjusted *p*-values). For transcript module 1 and peptide modules 4 and 8, the most significant overlapping GO terms include processes involving actin filament processes (“actin filament-based movement,” transcript module 1, adj. *p*-value 5.51e-05, “actin cytoskeleton organization,” peptides modules 4 and 8, adj. *p*-values 5.84e-02 and 4.11e-03 respectively), component assembly (“protein complex assembly,” transcript module 1, adj. *p*-value 1.09e-02, “cellular component assembly,” peptides module 4, adj. *p*-values 3.93e-07). It has been observed that SARS-CoV infection induces structural changes involving actin reorganization (Ng et al., 2004a,b; Surjit et al., 2004).

Transcript module 3 and peptide module 2 have a number of overlapping enriched GO terms including “nucleosome assembly” (transcript module 3, adj. *p*-value 9.66e-05, peptides module 2, adj. *p*-value 2.37e-05), and “protein-DNA complex subunit organization” (transcript module 3, adj. *p*-value 2.63e-03, peptide module 2, adj *p*-value 2.58e-04). Also, shared terms include “cellular component assembly” (transcript module 3, adj. *p*-value 5.48e-05, peptides module 2, adj. *p*-value 5.00e-05), and “cellular macromolecular complex assembly” (transcript module 3, adj. *p*-value 5.80e-05, peptides module 2, adj. *p*-value 4.71e-09). These functional associations again point to structural changes (Reghunathan et al., 2005).

In transcript module 4, peptide module 12 and peptide module 13, enriched GO terms associated with the regulation of processes. In particular, the “negative regulation of endopeptidase” (transcript module 1, adj. *p*-value 5.59-02, peptides modules 12 and 13, adj. *p*-values 8.36e-15 and 4.76e-06 respectively). Although, the transcript module is not highly significant when considering Bonferroni adjusted *p*-values, the GO term overlap between modules is strong here. Clearly endopeptidases, hydrolases, and cytokines have important roles in SARS-CoV infection (Loureiro and Ploegh, 2006). Cystatins, one of the represented protein families, has been proposed as a potential therapeutic target (Leung-Toung et al., 2006).

Overall, each set of integrated modules was overlapping in its functional annotation. Similarity in annotation between connected modules of different data types adds further evidence of true biological connection.

### Integration of module sub-graphs leads to richer results

A motivating use case for this approach was to develop a framework for integrated, network-based prioritization of targets for perturbation and validation. We compared the module level results to those attained by use of a naïve correlation-based method.

The naïve results took the form of two ranked lists for each data type. In the lists, there were both negative and positive correlations to the overall pathology phenotype. After mapping peptides and transcripts to Entrez IDs, correlation to the overall pathology phenotype, and the correlation between data types was compared (See Supplementary Figure 4). While some peptide-transcript pairs showed both strong correlation to the phenotype and strong correlation across data types, 33.1% of peptide-transcript pairs were essentially uncorrelated (across data type correlation, −0.1 < *r* < 0.1), and 4.6% of peptide-transcript pairs were anti-correlated (*r* < −0.3).

The naïve top ranked peptides and transcripts were not necessarily the most central within a given module. In transcript module 1, the most central (by correlation to the module eigenvector) was ranked 4th in the list of positive correlations. The next two top ranked module transcripts were not in the naïve top 10. On the peptide side, in module 4, the top ranked peptide was ranked 10th in the naïve list. This is due to the fact that the module construction is independent of any phenotype measurement. Module structure is a result of entities sharing a pattern of expression, rather than sharing a correlation with some external measurement.

The naïve results showed a reduction in significance compared to the module level tests. Four sets were taken from the ranked lists: 151 positively correlated peptides (PCP), 151 negatively correlated peptides (NCP), 130 positively correlated transcripts (PCT), and 130 negatively correlated transcripts (NCT). The results are listed in Supplementary Table 1. The PCP set of 151 peptides resulted in four significant GO terms, with Bonferroni adjusted *p*-values ranging from 2.67e-03 to 2.55e-02. The NCP set showed no significant GO term enrichment after Bonferroni adjustment. On the other hand, the PCT set of 130 transcripts showed 16 significant GO terms, with Bonferroni adjusted *p*-values in the range of 9.14e-11 to 2.24e-02. The NCT set showed three significant GO terms, Bonferroni *p*-value range of 2.61e-04 to 1.29e-02.

Two of the four significant GO terms found in the PCP set were also found in the PCT set (“protein polymerization” and “cellular macromolecular complex assembly”). However, one GO term, “cellular response to interleukin-4” (adj. *p*-value 2.67e-03), was not found in the enrichment results for both the naïve transcript sets and the peptide modules.

From the PCT set, eight of ten of the most significant GO terms were also found in the bipartite module graph results. The two that were not included were “regulation of actin filament length” (adj. *p*-value 1.07e-04) and “regulation of actin filament polymerization” (adj. *p*-value 3.96e-04). Enrichment in the NCT set showed one term that was also found in the bipartite module graph, and two terms, including “xenobiotic metabolic process” (adj. *p*-value 7.77e-03) and “response to xenobiotic stimulus” (adj. *p*-value 1.29e-02), which were not.

The module level organization provided more significance in enrichment tests compared to the naïve results. In peptide modules, the most significant results were found in peptide module 7 (not part of the module-graph) for GO terms “cellular component biogenesis” (adj. *p*-value 1.94e-17), “cellular macromolecular complex assembly” (adj. *p*-value 2.47e-17) and “nucleosome assembly” (adj. *p*-value 8.29e-12) where 15 of 36 Entrez IDs were present. In the NCP results, where the expression profile is negatively correlated with pathology, no GO terms were significantly enriched, whereas in the module-graph, there were sixteen significantly enriched GO terms.

**Figure 4:**
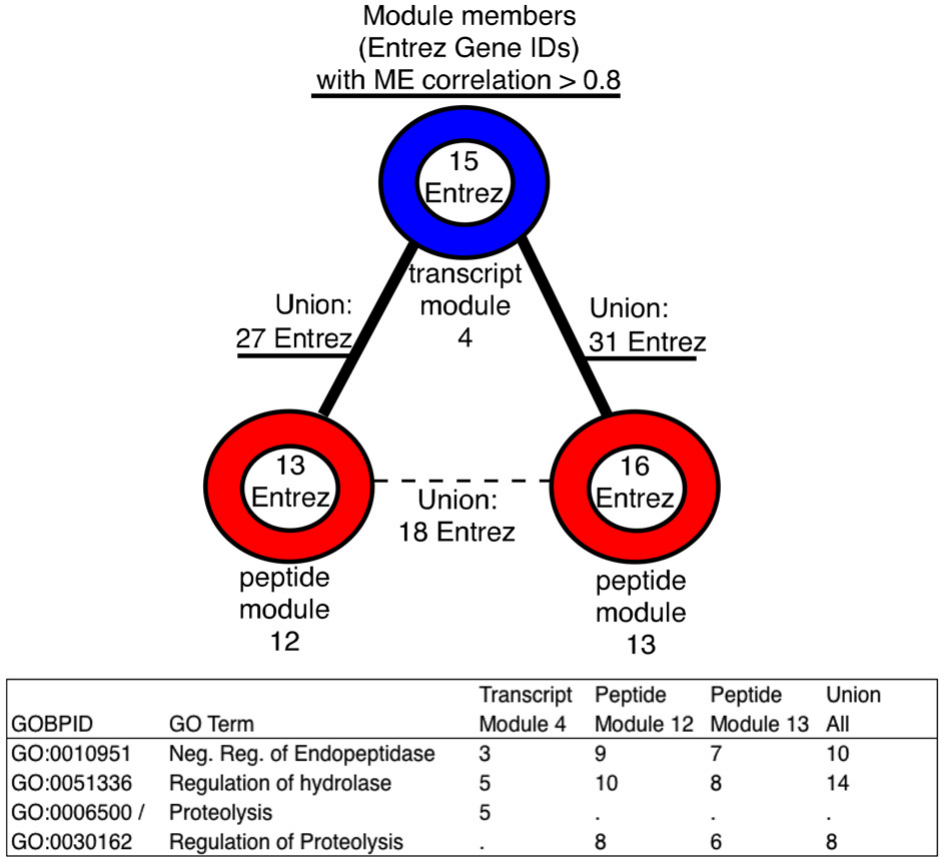
**An increase in the number of entities mapped to GO terms using the union of module members within a module sub-graph**. Enrichment was performed using only the module members with correlations to the module eigenvector greater than 0.8, these GO terms were all in the top 5 most significant after GO term enrichment within each module, and, after taking the union, they were still in the top 5 most significant. Adjusted *p*-values for all modules can be found in Supplementary Table 1.

On the module level, highly ranked members (by module centrality), showed little overlap across data types in terms of shared Entrez IDs, even in the presence of significant module membership overlap (Supplementary Tables 1 and 2). Regardless, we found that significant GO term enrichment was observed to be overlapping across the data types. By utilizing the module sub-graph, the most central members were functionally compared, producing potentially novel connections to investigate. An example was found in the module sub-graph including transcript module 4 and peptide modules 12 and 13 (See Figure 4). The most central members (*r* > 0.8) enriched the same GO terms, and in taking the union across modules, each data type brings unique, and functionally related, members to the analysis.

## Discussion

In this work, a new strategy for data integration has been developed that leverages existing network inference methodology for transcriptomics and more recent extensions in proteomics. The method was applied to infection time course data to examine both the individual networks and the bipartite module graph with regard to phenotypic correlation and functional enrichment, as well as to provide integrated prioritization across data types.

Information needed to construct the bipartite module graph included membership overlaps, summary eigenvector correlations and common correlations to phenotype. The strong correlations between module eigenvectors highlights the biological organization observed across data types. Given that a large portion of peptides and transcripts, mapping to the same Entrez ID, are essentially uncorrelated, it is striking to observe the modularity in our inferred networks, and the strong connection between a subset of the modules. Effectively, this can provide the foundation of a true multi-omic signature of SARS-CoV viral infection that may have relation to other viral respiratory infections as well. Additionally, this provides a perspective on modularity in the proteome and its relationship to the module structure of the transcriptome.

Initially modeling each data type individually offers a high degree of flexibility in the analysis. In co-expression network construction, one has choices about correlation type or metric for association between nodes in the network, how or whether to scale the associations, and how to cluster for module discovery. Additionally, there are questions of normalization and missing data that can affect on the downstream network structure. These parameters can be separately tuned in order to produce optimal independent networks on data types, which can be used to produce integrated module graphs. Generating well-formed, independent networks should improve the odds of successful integration since they each more accurately reflect the underlying biology. Without treating different data sources independently, it is possible that the inherent noise found in biological data would obscure patterns linking data types.

In the correlation of expression profiles across data types, there was a large degree of uncorrelated peptides and transcripts, which has been previously observed (Ghazalpour et al., 2011). Additionally, there are peptides and transcripts, which are both correlated to phenotype, but are anti-correlated across data types. The apparent disconnect between data types makes interpretation difficult. The naïve ranked list is expected to be less ordered compared to within-module rankings. This is seen in the fact that for NCP, the naïve method returned no significant enrichment results, while the module level results did show significance. Compared to the naïve ranking approach, the module level analysis avoided the problem of uncorrelated peptides and transcripts, by connecting modules using a set of metrics, rather than simple Entrez ID mapping. Additionally, the module organization returned considerably more significant enrichment results, and also showed more functional overlap across data types.

One of the most difficult aspects of data integration can be the annotation of highly heterogeneous data sets, connecting the transcript and peptide to their correct source gene for instance. For transcripts, this annotation is more straightforward since microarray probes have been designed specifically to avoid degeneracy among genes and have relatively good documentation. On the other hand, given a peptide, it can be quite difficult to determine what gene it ultimately resulted from. Our knowledge of the proteome is still rapidly expanding, directly affecting our peptide-transcript integration solution. As proteomics technologies improve, however, the intersection between the proteome and transcriptome will continue to grow, improving our integrated models, and our understanding of the cell.

In this work, we have provided a strategy for integrated analysis in order to shed light on complex biology. With these methods, it is possible to learn novel and biologically relevant information about the relationship between the host and pathogen, but more generally between the transcriptome and the proteome. This work should prove to be platform independent, allowing the use of RNAseq (see Iancu et al., 2012b) or other forms of proteomic data. This can then be used to inform systems-level prioritization for the subsequent perturbation and validation experiments, allowing the full realization of systems based approaches.

## Author contributions

David L. Gibbs designed the methods, analyzed the results, developed the code, and wrote the manuscript. Lisa Gralinski and Ralph S. Baric are responsible for all aspects of generating the SARS data. Shannon K. McWeeney designed methods, contributed to the analysis and edited the manuscript.

## Funding

This work was supported by the National Institute of Allergy and Infectious Diseases, National Institutes of Health, Department of Health and Human Services [5U54AI081680, U19AI100625] and National Library of Medicine [3T15LM7088-18S1].

### Conflict of interest statement

The authors declare that the research was conducted in the absence of any commercial or financial relationships that could be construed as a potential conflict of interest.

## Supplementary material

Supplementary Table 1 **Shows the top ten enriched GO terms for each module sub-graph.** The tables correspond to the module sub-graphs in Figures 1, 3, and 4. Also contains the GO enrichment results for the Naïve comparison.

Supplementary Table 2 **Shows the top ten entities by correlation with the module summaries (ME, module eigenvector).** The second column shows the correlation with the Overall-total-score phenotype describing the level of lung pathology. The tables correspond to the module sub-graphs in Figures 1, 3, and 4.

Supplementary Table 3 **The mouse phenotype for experiment SM001, for both mocks and infected mice.** Pathological variables are given along with viral dosages (Dose), day of tissue sampling post infection (Day), Mouse ID (RepName).

Supplementary Figure 1 **Pearson correlations between transcript module eigenvector summaries and mouse phenotypes.** In rows, each module is labeled as ME_followed by the module ID number.

Supplementary Figure 2 **Pearson correlations between peptide module eigenvector summaries and mouse phenotypes.** In rows, each module is labeled as ME_ followed by the module ID number.

Supplementary Figure 3 **Pearson correlations between peptide module eigenvector summaries and transcript module summaries.** Each module is labeled as ME_ followed by the module ID number.

Supplementary Figure 4 **Points are composed of peptide-transcript pairs, where each entity maps to the same Entrez ID.** The x and y axis are correlations with the overall pathology phenotype, while the size and hue indicates correlation across the data types. Blue indicates a positive correlation while red shows a negative correlation.

**Transcript_Module_Contents**. A large table with columns: Probe ID (the transcript probe), Module (the assigned module), MM1 to MM6 (correlations with module eigenvectors), p.MM1 to p.MM6 (*p*-values for each correlation), and then correlations with phenotype variables, and *p*-values for phenotype correlations.

**Peptide_Module_Contents**. A large table with columns: Peptide ID, Module (the assigned module), MM1 to MM14 (correlations with module eigenvectors), p.MM1 to p.MM14 (*p*-values for each correlation), and then correlations with phenotype variables, and *p*-values for phenotype correlations.

**Supplementary_Network_Results**. A brief account of peptide and transcript networks in terms of module sizes and module-significance permutation tests. Also shown are module overlap sizes with permutation test results, and the pairwise module prioritization results for each phenotype.
