# Supplementary material for: Multi-omic network signatures of disease
Source: Front Genet. 2014 Jan 7;4:309. doi: 10.3389/fgene.2013.00309 (PMC3882664; doi:10.3389/fgene.2013.00309)
Supplement: Supplementary Table 1 — Shows the top ten enriched GO terms for each module sub-graph. The tables correspond to the module sub-graphs in Figures 1, 3, and 4. Also contains the GO enrichment results for the Naïve comparison. [file DataSheet1.ZIP › Supplementary_Information/Supplementary_Figure_1.pdf]

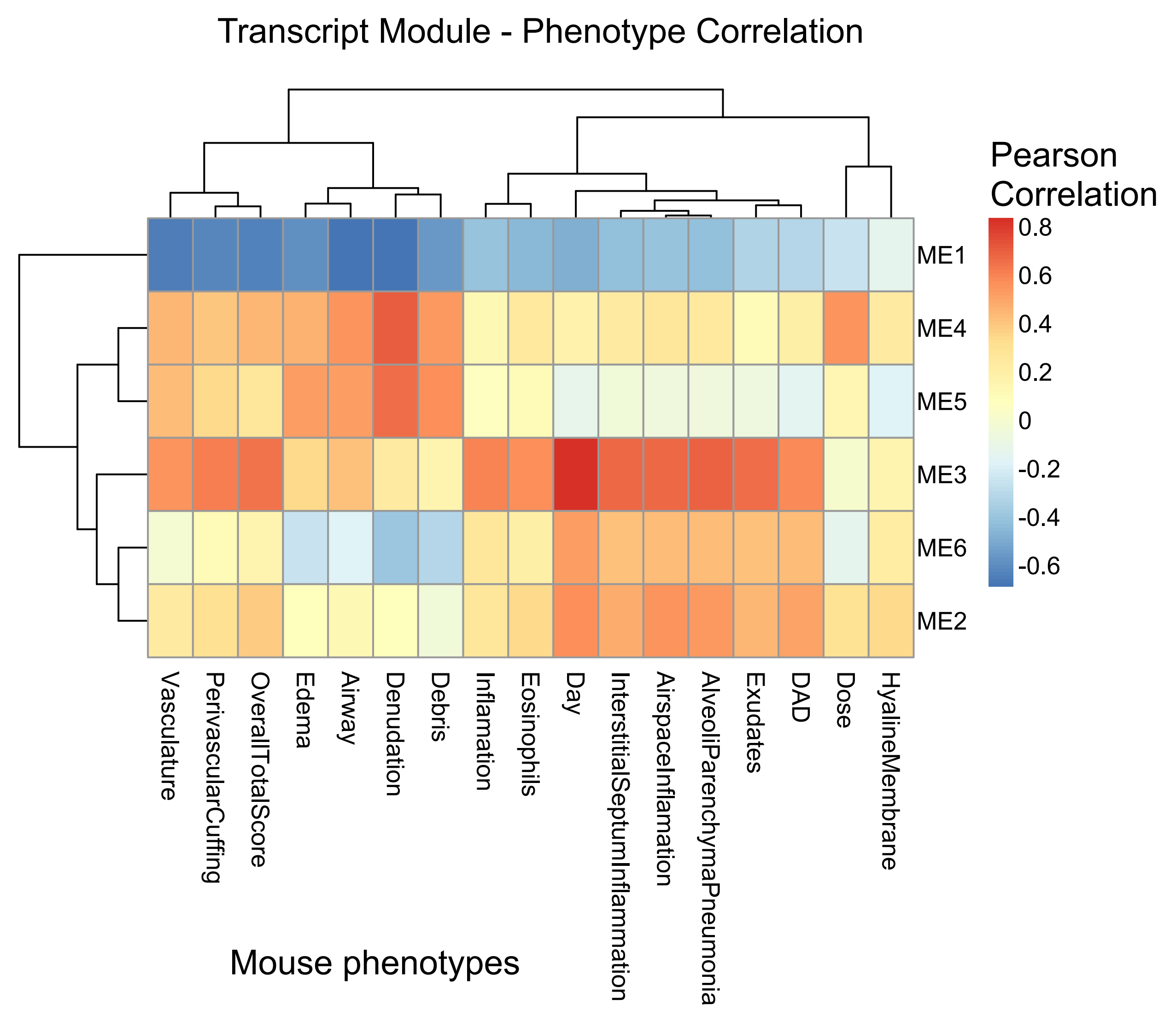
Transcript Module - Phenotype Correlation
Pearson Correlation
0.8
0.6
0.4
0.2
0
-0.2
-0.4
-0.6
ME1
ME4
ME5
ME3
ME6
ME2
Vasculature
PerivascularCuffing
OverallTotalScore
Edema
Airway
Denudation
Debris
Inflamation
Eosinophils
Day
InterstitialSeptumInflammation
AirspaceInflamation
AlveoliParenchymaPneumonia
Exudates
DAD
Dose
HyalineMembrane
Mouse phenotypes
