# Supplementary material for: Multi-omic network signatures of disease
Source: Front Genet. 2014 Jan 7;4:309. doi: 10.3389/fgene.2013.00309 (PMC3882664; doi:10.3389/fgene.2013.00309)
Supplement: Supplementary Table 1 — Shows the top ten enriched GO terms for each module sub-graph. The tables correspond to the module sub-graphs in Figures 1, 3, and 4. Also contains the GO enrichment results for the Naïve comparison. [file DataSheet1.ZIP › Supplementary_Information/Supplementary_Figure_4.pdf]

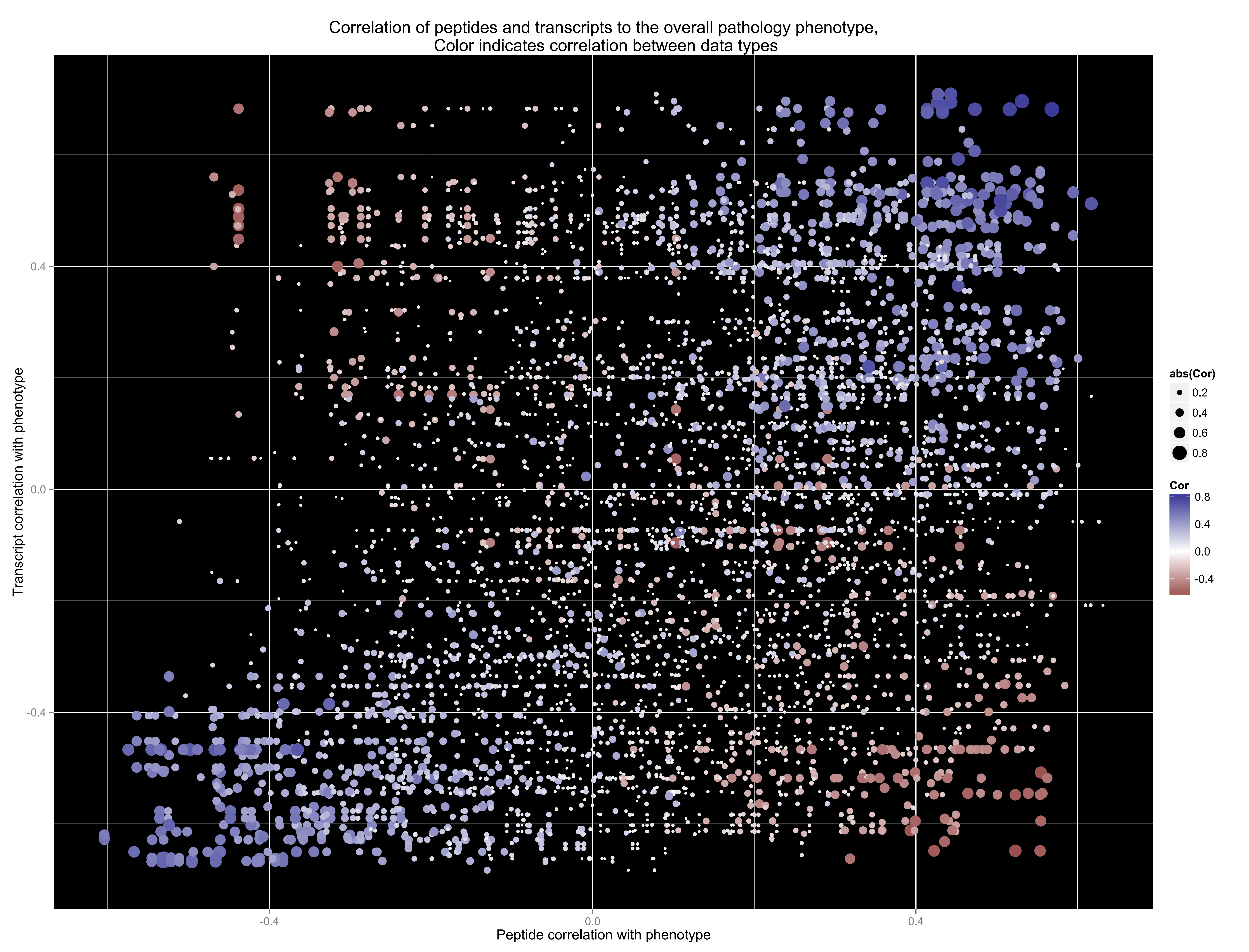
Correlation of peptides and transcripts to the overall pathology phenotype,
Color indicates correlation between data types
Transcript correlation with phenotype
Peptide correlation with phenotype
0.4
0.0
-0.4
-0.4
0.0
0.4
abs(Cor)
0.2
0.4
0.6
0.8
Cor
0.8
0.4
0.0
-0.4
