# Supplementary figures and images for: Multi-omic network signatures of disease
Source: Front Genet. 2014 Jan 7;4:309. doi: 10.3389/fgene.2013.00309 (PMC3882664; doi:10.3389/fgene.2013.00309)

Peptide module-phenotype correlations.

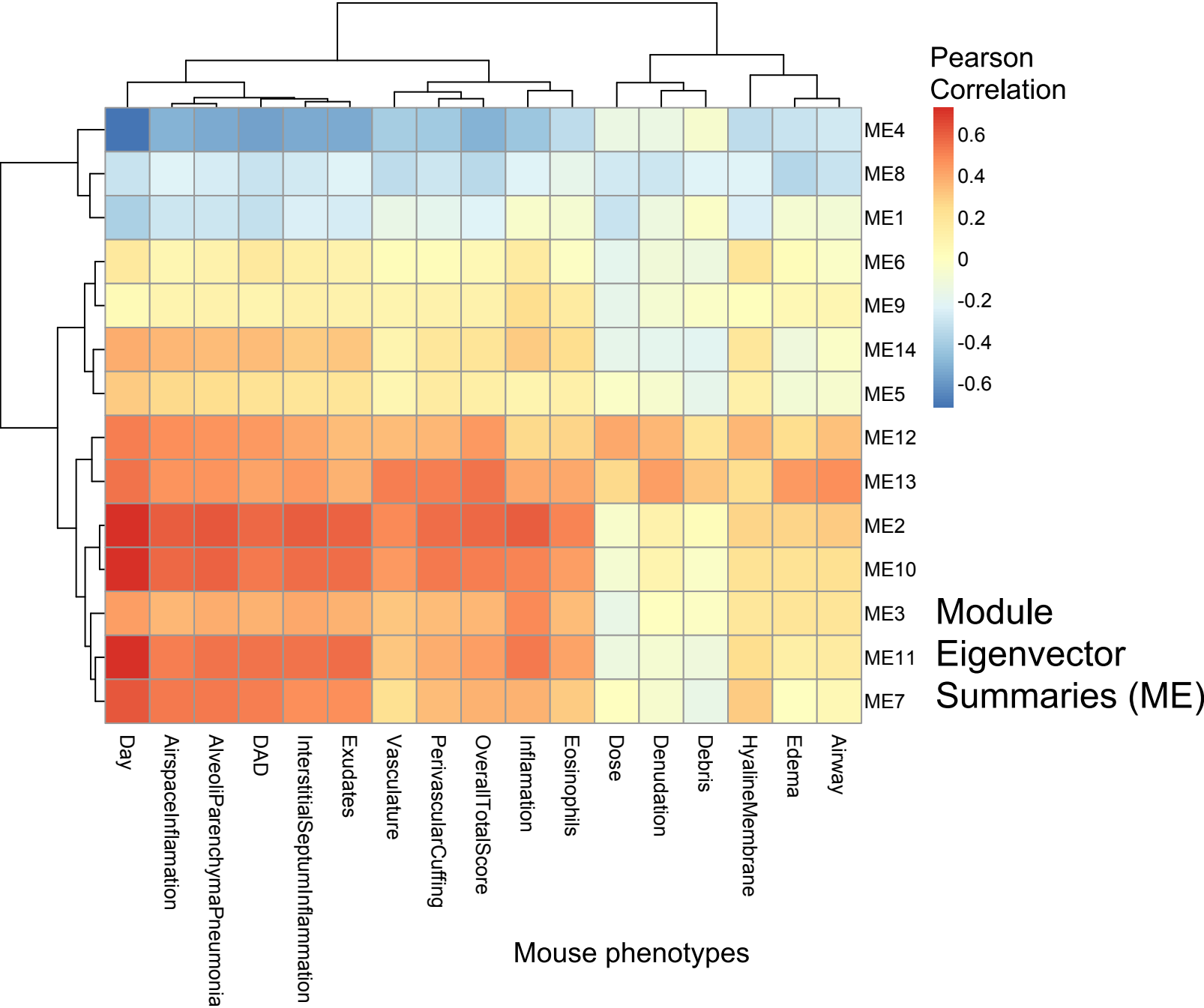

Supplement: Supplementary Table 1 — Shows the top ten enriched GO terms for each module sub-graph. The tables correspond to the module sub-graphs in Figures 1, 3, and 4. Also contains the GO enrichment results for the Naïve comparison. [file DataSheet1.ZIP › Supplementary_Information/Supplementary_Figure_2.pdf]

# Peptide-Transcript module correlations.

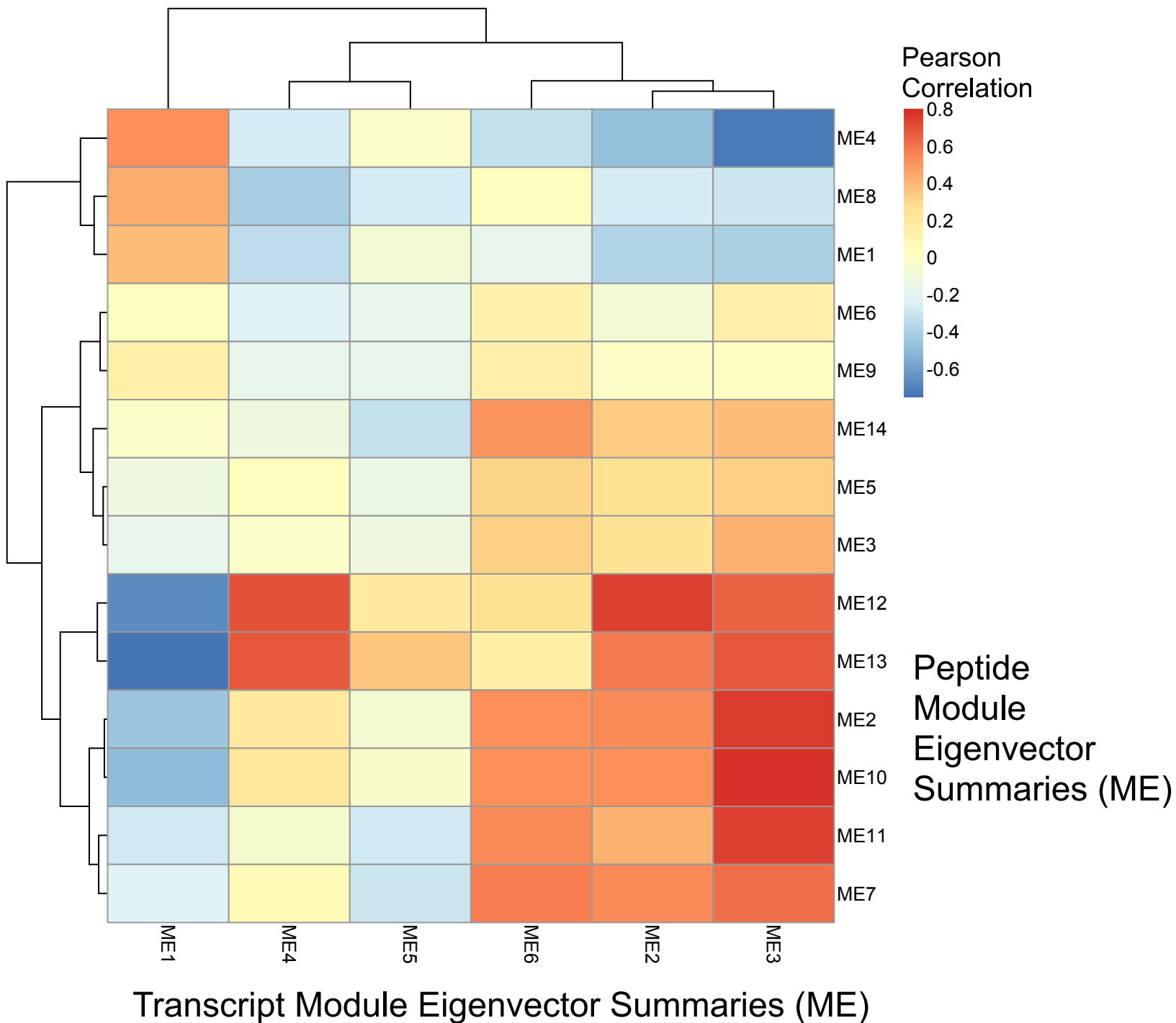

Supplement: Supplementary Table 1 — Shows the top ten enriched GO terms for each module sub-graph. The tables correspond to the module sub-graphs in Figures 1, 3, and 4. Also contains the GO enrichment results for the Naïve comparison. [file DataSheet1.ZIP › Supplementary_Information/Supplementary_Figure_3.pdf]
